# Vitamin D and Health: Establishing Causal Relationships Through Observational Evidence and Hill’s Criteria in a Biological Framework

**DOI:** 10.3390/nu18172902

**Published:** 2026-09-04

**Authors:** William B. Grant, Sunil J. Wimalawansa

**Affiliations:** 1 Sunlight, Nutrition, and Health Research Center, 1745 Pacific Ave., Ste. 504, San Francisco, CA 94109, USA; 2 Endocrinology & Human Nutrition, Department of Medicine, Cardiometabolic & Endocrine Institute, North Brunswick, NJ 08902, USA; suniljw@hotmail.com

**Keywords:** cancer, blood pressure, cardiovascular disease, COVID-19, dementia, diabetes mellitus, multiple sclerosis, periodontal disease, vitamin D, UVB

## Abstract

Vitamin D has been linked to beneficial effects across more than 100 health outcomes, generating substantial interest in its extraskeletal functions. However, because nutrients differ fundamentally from pharmaceuticals in biology and mechanisms of action, conventional pharmaceutical-style randomized controlled trials (RCTs) are often inappropriate for evaluating nutrient efficacy. Instead, well-designed observational studies that have assessed serum 25-hydroxyvitamin D [25(OH)D] concentrations and correlated them with biologically relevant clinical outcomes provide evidence of effectiveness and establish causality. Since vitamin D primarily acts as a preventive agent for chronic diseases, the strongest evidence is derived from large, long-term, ecological and prospective cohort studies. In contrast, many RCTs have failed to demonstrate significant benefits because of methodological limitations, including enrollment of vitamin D-sufficient participants, inadequate dosing regimens, vitamin D supplementation in control groups, short intervention periods, and analyses based on assigned dose rather than serum 25(OH)D concentrations achieved, which determine biological responses. Early ecological studies demonstrated inverse associations between solar ultraviolet B doses and the risks of cancer, cardiovascular disease (CVD), and infectious diseases. These findings have been consistently supported by numerous prospective larger cohort studies linking higher serum 25(OH)D concentrations with improved health outcomes. Observational studies also offer important advantages, including being large and covering diverse populations, wide variations in vitamin D status, longer follow-up, and adjustment for multiple confounding factors. When interpreted within a biological framework using Bradford Hill’s criteria for causality—including consistency, strength and temporality of associations, dose–response relationships, biological plausibility, mechanistic evidence, coherence, and experimental support—the accumulated evidence strongly supports causal relationships that vitamin D sufficiency reduces risks of several diseases. This narrative review critically evaluates the evidence for cancer, CVD, blood pressure, COVID-19, type 1 and type 2 diabetes, periodontal disease, multiple sclerosis, and dementia. For these diseases, the authors concluded that the evidence satisfies Hill’s criteria for causality and aligns with current understanding of vitamin D biology and nutrient–health relationships.

## 1. Introduction

Vitamin D is a secosteroid that has been studied extensively regarding its role in maintaining health and reducing risks and severity of a variety of diseases. Before 2000, vitamin D research was generally limited to skeletal research. Since the early 2000s, interest has turned to the extra-skeletal system benefits of vitamin D, which continues. As of 27 August 2026, there were 91,799 entries at https://pubmed.ncbi.nlm.nih.gov/ (accessed 27 August 2026) with vitamin D in the title or abstract, of which 77,327 were published after 1 January 2020. The most frequent type of clinical research related to vitamin D in observational studies. Observational studies include ecological studies, both geographical and temporal, as well as cross-sectional, prospective cohort, case-control, and nested case-control studies. Such studies are generally considered unable to be used to establish causal relationships for pharmaceutical agents, but not so for micronutrients [1].

Randomized controlled trials (RCTs) are conducted either due to misunderstanding this fundamental principle or to illustrate pharmaceutical biases against micronutrients, like vitamin D. Therefore, it is unsurprising that (see below) most vitamin D RCTs have failed to support the role of vitamin D in reducing risk of disease incidence, severity, mortality, or beneficial effects in treating diseases. (When not stated, it should be assumed that supplemented vitamin D refers to vitamin D_3_ unless otherwise noted.) These findings were primarily due to the use of RCT-based study designs that were specifically designed for synthetic pharmaceuticals, which are unsuitable for nutrients [1,2]. This situation strongly suggests that observational studies should be used for evaluating nutrients and for establishing causal relationships. An approach that has been used is applying Hill’s criteria for causality in a biological system [3].

This narrative review will examine the following topics. First, RCTs will be considered, reference to how they are supposed to be conducted, how they conducted for pharmaceutical drugs, how are they conducted for vitamin D, and how should they be conducted for vitamin D. Second, observational studies will be examined in detail including how they are conducted and the effect of “regression dilution” in prospective cohort studies (PCS) and how confounding factors are handled. Available databases for further studies are identified. Advantages of observational studies will be discussed.

Third, this review will examine how Hill’s criteria, as applied to vitamin D, will be discussed specifically. An index search identified 20 representative and relevant studies, of which over half were selected for detailed review. They were tabulated, summarized, analyzed, and evaluated for evidence supporting or refuting the key criteria for causality. The conditions reviewed include cancer, cardiovascular disease (CVD), hypertension/blood pressure (BP), COVID-19, type 1 diabetes mellitus (T1DM), type 2 diabetes mellitus (T2DM), periodontal disease, multiple sclerosis, and dementia. Overall, the goal of this review is to examine whether vitamin D is causally linked to maintaining good health and reducing disease risk and severity, to inform updates to disease policies and vitamin D health policies.

### 1.1. Vitamin D and the Expanding Science of Disease Prevention

Vitamin D has emerged as one of the most biologically pleiotropic micronutrients in human physiology. It is available as a highly cost-effective intervention with effects that extend far beyond its canonical role in skeletal health [4]. Active vitamin D signaling is mediated through various mechanisms, including vitamin D receptor-DNA interactions (genomic actions), local signaling pathways present in peripheral target cells such as intracrine and paracrine actions, and its direct actions on membranes [5,6]. These mechanisms support its involvement in cellular differentiation, immune regulation, inflammation, endocrine function, and other mechanisms relevant to disease prevention [7]. This broad tissue-level distribution helps explain why vitamin D has been linked in the literature to a wide range of disorders and clinical outcomes, including musculoskeletal disorders, cardiometabolic disease, autoimmune conditions, infections, and selected malignancies [4,7].

Most of the effects of vitamin D are mediated through the most active vitamin D metabolite, 1,25-dihydroxyvitamin D (calcitriol), a hormonal metabolite 1,25-dihydroxyvitamin D (calcitriol) binding to a vitamin D receptor attached to the nucleus in a cell and modulates gene expression [8]. As reported in 2019, following supplementation of 30 healthy adults with 600, 4000, or 10,000 IU/d of vitamin D_3_ for 6 months, there were alterations in gene expression with 162, 320, and 1289 genes, respectively—up- or down-regulated—in their white blood cells [9]. This study suggests the importance of maintaining higher doses and 25(OH)D concentrations.

Yet despite the expanding evidence base, vitamin D remains underappreciated in routine clinical practice, in part because its effects are subtle yet complex, context-dependent, and difficult to assess in individual patients, and in part since RCTs have largely failed to support its health benefits. Its potential value is often minimized in medical education at every level and clinical decision-making, where focus tends to remain on symptomatic treatment rather than upstream prevention and biologic optimization. Consequently, vitamin D’s cost-effectiveness as a preventive strategy is substantially underestimated, even though its accessibility, safety profile, and potential to influence multiple disease pathways make it an especially compelling subject for preventive medicine and public health research. As highlighted in this review, vitamin D is compelling to include not only in infectious and autoimmune disease prevention but also in chronic disease prevention and alleviation, and in clinical practice and public health protocols, yet none currently exist.

### 1.2. Healthy Vitamin D Doses and Serum 25(OH)D Concentrations

Contemporary vitamin D research shows that fixed-dose supplementation is inadequate because individuals reach very different serum 25(OH)D concentrations on the same intake. A team of global experts convened by Grassroots Health Nutrient Research Institute (https://www.grassrootshealth.net/ (accessed 21 May 2026)) and the Institute for Pure and Applied Knowledge (https://ipakedu.online/) emphasized that vitamin D guidance must prioritize achieved blood levels rather than uniform dosing (manuscript in preparation) serum 25(OH)D concentrations between 40–80 ng/mL appear to support the widest range of physiological functions, with no adverse effects, while levels below 30 ng/mL remain deficient and increase risks for diseases [10,11]. Weight-based dosing formulas—such as those proposed [12]—offer a practical method to achieve and maintain consistent therapeutic levels (across diverse body sizes and metabolic profiles). These principles align with emerging evidence showing that vitamin D status, not intake alone, determines biological effectiveness [13].

Vitamin D biology also depends on cofactors such as magnesium and vitamin K_2_, which influence metabolism, receptor activation, and calcium handling [14,15,16]. Variability in genetics, absorption, adiposity, medications, and chronic disease [17] further contributes to differences in serum response, underscoring the importance of measuring 25(OH)D when possible and adjusting supplementation accordingly [12,17,18]. Maintaining optimal serum levels may support immune regulation, metabolic health, and chronic disease prevention, while toxicity remains rare at physiologic concentrations [19].

Historically, serum 25(OH)D concentrations of approximately 20 ng/mL have been considered sufficient to prevent overt skeletal disease in most healthy individuals [20]. Other organizations, like the Endocrine Society 2011, have recommended a minimum threshold of 30 ng/mL, particularly for populations at increased risk of deficiency [21]. Laboratory reference ranges are often seen at 30–100 ng/mL. However, in recent years, many vitamin D clinical researchers and clinicians have been recommending higher target ranges, typically between 40 and 60 ng/mL or 40 and 80 ng/mL. Even higher targets may be chosen to acknowledge individual variability and may be appropriate for selected populations or specific clinical circumstances when monitored appropriately.

More recent clinical studies have shown that maintaining serum 25(OH)D concentrations of 40–80 ng/mL, optimally above 50 ng/mL, may maximize vitamin D-mediated health benefits without increasing adverse effects. This range has been estimated to support approximately 99.8% of vitamin D-responsive physiological functions and disorders. Examples of studies that support conditions, disorders, and diseases that minimum levels of 40 ng/mL benefit include (A), Pregnancy -related (pre-eclampsia [22], premature births [23,24], Cesarean sections [25], Gestational diabetes [26], conversion of prediabetes to diabetes [27], multiple sclerosis [28], autoimmune disorders [29], and cancer [30,31,32].

Several recent independent research groups indicate that serum 25(OH)D concentrations associated with the greatest protection against diseases like severe infections are above 50 ng/mL, rather than the commonly cited thresholds of 20–40 ng/mL. In particular, landmark studies by Quraishi and colleagues involving critically ill and surgical patients identified an inflection point at approximately 50 ng/mL, above which immune function was enhanced, and the risk of severe infections and sepsis was significantly reduced [33].

Given the absence of evidence of adverse effects within the 40–80 ng/mL range, maintaining serum 25(OH)D concentrations of at least 50 ng/mL may be an effective strategy for optimizing innate immune function and reducing the risk of severe infections, intensive care admission, and sepsis. For example, the COVID-19 pandemic provided substantial evidence of the clinical relevance of adequate vitamin D status in infectious disease outcomes. More than 100 small, independent RCTs published during the COVID-19 era provided evidence that higher serum 25(OH)D concentrations at the time of SARS-CoV-2 infection were associated with less severe and less symptomatic disease, as well as fewer hospitalizations, ICU admissions, and deaths [34,35].

Thus, maintaining physiological serum 25(OH)D concentrations between 50 and 80 ng/mL may offer greater protection than accepting the customarily recommended lower levels, which may be associated with less complete biological responses. Importantly, there is no evidence of additional risk within this range.

## 2. Methods and Study Types Examined

This is a narrative review examining causal relationships through observational study evidence and Hill’s Criteria in a Biological Framework. The objective was to examine the causal relationships between vitamin D and the above-mentioned disorders using published prospective, large clinical studies.

The journal literature was searched using Google Scholar, MEDLINE, EMBASE, and PubMed.gov. Both historically important and recent publications were sought that were published in English from 1991 through June 2026 related to the topic [36]. Evidence was prioritized based on relevance, methodological quality, and consistency [37]. A broad review approach was adopted; citations were screened for relevance, data were synthesized, categorized into subtopics, and hierarchical tables were created. Additional evidence was drawn from peer-reviewed sources, web-based reports, and grey literature when needed, each assessed for credibility, biases, and relevance [38,39].

To search for representative examples of using Hill’s criteria to demonstrate causal dietary factors-disease relationships, the searches used “Hill’s criteria” and names of diseases that affect large fractions of populations. For the present understanding of these diseases, the search string included the disease name and words related to the identified risk factor.

For Hill’s criteria reviews, the search string was “Hill’s criteria, vitamin D”. Publications considered for inclusion had to deal with serum 25(OH)D concentrations. When there were multiple publications over a long period, those that made important additions to the analysis were chosen. Recent increases in understanding the role of vitamin D for the diseases discussed were sought by searching “vitamin D” along with the name of the disease.

## 3. Results

### 3.1. Randomized Controlled Trials

First, we discuss why RCTs have had limited effect in finding that vitamin D supplementation has health benefits. They have generally been based on guidelines for pharmaceutical drugs, not nutrients. Heaney’s guidelines for RCTs of nutrients should be followed [40]. RCTs are generally considered the “gold standard” for establishing causal relationships for synthetic pharmaceutical agents. Their biology and dose–response relationships are much different from micronutrients, like vitamin D, which is a threshold and network nutrient [1]. RCTs generally enroll participants, randomly assign about half of the participants to treatment while the controls receive a placebo (or a comparator). The RCT is conducted for an extended period, and both beneficial and adverse health effects are noted. At the conclusion, comparisons are made for those treated vs. controls.

When conducting RCTs for pharmaceutical drugs as they were intended for, several steps are taken to maximize the likelihood of demonstrating efficacy while minimizing adverse effects. First, preliminary studies determine doses that provide benefits without toxicity. Second, participants are selected who tolerate the medication and are most likely to benefit from the intervention. Third, an appropriate placebo is chosen that is expected to have minimal effects on outcomes; however, some placebos may exert beneficial effects, requiring comparisons with established therapies. Fourth, sample size and study duration are calculated to achieve statistical significance, typically defined as *p* < 0.05. Fifth, investigators are recruited and incentivized to enroll participants retained during the study. Sixth, investigators submit results to the sponsoring company, which screens and combines data and often prepares the manuscript, injecting biases.

In the pharmaceutical industry, investigators conduct RCTs; although investigators may be listed as coauthors, they are generally not provided with full access to detailed analyses and may not have access during the write-up of the final manuscript [41]. Thus, while each step is designed to demonstrate beneficial effects, biases and limited transparency in data analysis and false claims may increase the risk of inappropriate interpretations [42]. This concern may partly explain why Ioannidis concluded in 2005 that many published research findings are false [4]. It suggested that instead of relying on statistical significance, researchers should improve understanding of the range of true relationships (R values)—positive predictive values and the pre-study odds [43,44].

Most vitamin D RCTs have failed to demonstrate significant health benefits of supplementation (see [45,46]). A major reason may be that their designs have been based largely on pharmaceutical drug trial models rather than nutrient-specific principles [1,8]. In pharmaceutical trials, the control group does not receive the drug being tested. In contrast, vitamin D is a nutrient that all individuals obtain through endogenous synthesis from exposure to solar UVB, dietary sources, and supplements. Therefore, withholding vitamin D is not equivalent to withholding a pharmaceutical agent. Hill’s criteria have been used to evaluate causal dietary factor-disease relationships for several diseases, as shown in Table 1.

Robert Heaney proposed guidelines for nutrient RCTs in 2014 [40]. These include establishing the nutrient dose–response relationship, measuring baseline nutrient status, and enrolling primarily participants with insufficient levels who are most likely to benefit. The intervention should be sufficient to raise nutrient concentrations into the range associated with improved outcomes. Additional considerations include ensuring adequate levels of interacting nutrients, monitoring nutrient concentrations during the trial to confirm adherence and biological response, and verifying that the intended target levels are achieved. Importantly, for vitamin D trials, serum 25(OH)D concentration—not the administered vitamin D dose—is the relevant biological variable for assessing associations with clinical outcomes [1,8].

In addition to recruiting those with sufficient nutrients into clinical trials, the main limitations in the design of many vitamin D RCTs relate to failure to evaluate the relationship between achieved serum 25(OH)D concentrations and health outcomes. Most observational studies show that disease risk rises sharply when 25(OH)D concentrations fall below approximately 20 ng/mL (i.e., deficiency), whereas additional increases above this level (especially beyond 30 ng/mL) are associated with slower changes in risk. However, many large vitamin D RCTs enroll participants with baseline 25(OH)D concentrations already above 25–30 ng/mL, as in the case of the VITAL study [60], inherently limiting the potential to demonstrate benefit.

Furthermore, control groups in many US and European trials (inappropriately) receive vitamin D supplementation (approximately 400 IU/day), based on historical recommendations for rickets prevention in children [61]. In the United States, participants are also commonly allowed an additional 600–800 IU/day from over-the-counter supplements, consistent with Institute of Medicine (IoM) recommendations [20]. 800 IU/day can increase serum 25(OH)D by approximately 5 ng/mL (12 nmol/L [62] (early part of the dose–response curve), and participants also obtain vitamin D from diet and sunlight exposure; those in the control groups often maintain 25(OH)D concentrations above deficiency levels.

Consequently, differences in vitamin D status between intervention and control groups become minimized to a level of clinical insignificance, reducing the ability of RCTs to detect meaningful health benefits. As a result of including most participants with 25(OH)D concentrations above 20 ng/mL, it is unsurprising that many vitamin D RCTs have failed to find beneficial effects: [1,45,46,60,63,64,65,66]. As stated in a 2022 review, “In conclusion, supplementation of vitamin D-replete individuals does not provide demonstrable health benefits. This conclusion does not contradict older guidelines that severe vitamin D deficiency (VDD) should be prevented or corrected” [67].

Those who are likely to benefit most from vitamin D supplementation are those with VDD. The authors searched Google Scholar and PubMed.gov for representative studies of the prevalence of VDD in national populations. Representative study results are given in Table 2. The lowest rate of VDD is in Australia. This country has high solar UVB doses and a population of largely Fitzpatrick skin type 1 due to its Celtic origin. The country with the highest rate of VDD is China.

The Consensus statement from the Second International Conference on Controversies in Vitamin D [80] noted that RCTs with results reported as of 2019 did not evaluate the effect of VDD on non-skeletal health outcomes [80]. This finding explains the finding by Autier that most vitamin D RCTs did not find beneficial effects of vitamin D supplementation for non-skeletal effects [45]. The situation did not change after the publication of three larger vitamin D RCTs: VITAL in the US [60] ViDA in New Zealand [81] or D-Health in Australia [65]: all three of them had major study design errors [1,2].

Notably, “there was no interaction between randomization group and baseline measured or predicted serum 25(OH)D concentration in VITAL, ViDA, or D-Health, but in all three trials only a few participants had 25(OH)D concentration less than 50 nmol/L.” [65]. ViDA included 5110 community-dwelling participants whose baseline 25(OH)D concentrations were measured in 2011 or 2012 [81]. Thirty-one of the participants had baseline 25(OH)D < 10 ng/mL, and 1176 had baseline 25(OH)D between 10 and 20 ng/mL out of 5110 subjects. Of the twelve outcomes reported, only four were significantly different for those with baseline 25(OH)D < 20 ng/mL: bone mineral increase, lung function, and arterial function.

In RCTs designed for pharmaceutical drugs, one of the most important criteria for inclusion is that the participants have a reasonably high risk of an adverse health event that the drug is expected to prevent or treat. Applying this criterion to vitamin D means that participants should be vitamin D deficient; otherwise, there is no reason to conduct that trial [1].

In contrast, here are two examples from observational studies showing the importance of having large numbers of vitamin D-deficient participants. A vitamin D supplementation study was conducted in Iran with pregnant women [26]. A total of 900 pregnant women at each of two maternity hospitals were included. At baseline, the mean 25(OH)D concentration at each hospital was near 11 ng/mL. Pregnant women at the non-screening site were monitored but not supplemented with vitamin D. Those at the screening site were supplemented with sufficient vitamin D to try to increase serum 25(OH)D above 20 ng/mL (i.e., sufficient for skeletal health). At delivery, the median [interquartile range (Q1–Q3)] was 11 (8–71) ng/mL, while at the screening site it was 21 (18–25) ng/mL. The results of screening were reduction in risk of preeclampsia by 60% (OR = 0.40 [95% CI, 0.30–60]), number needed to screen (NNS) to reduce one case was 11 (95% CI, 8–17), reduction in risk of gestational diabetes by 50% (OR = 0.50 [95% CI 0.34–0.88]), NNS 50 (95% CI, 2–167), and preterm delivery, by 40%, NNS 20 (95% CI, 13–50).

Another observational study of the effect of vitamin D supplementation on the risk of CVDs—myocardial infarction and all-cause mortality rate gives some indication of the number of participants with VDD required [82]. The study was based on patients of the Veterans Health Administration receiving care from 1999 to 2018 [82]. The study was based on 20,025 patients with 25(OH)D concentrations < 20 ng/mL at first measurement. The patients were divided into three groups: those who were not prescribed vitamin D supplements (Group A), those who were prescribed vitamin D supplements and achieved 25(OH)D concentrations of 21–29 ng/mL at follow-up (Group B), and those who were prescribed vitamin D supplements and achieved >30 ng/mL at follow-up (Group C). A propensity-matched comparison of those in Group C (*n* = 2942) vs. those in Group A (*n* = 10,014) found an HR for myocardial infarction = 0.73 (95% CI, 0.55–0.96). The same analysis for Group C (*n* = 3085) vs. Group A (*n* = 5266) found HR = 0.65 (95% CI, 0.49–0.85). This example shows the importance of having many participants with VDD for studies of vitamin D and CVD.

Another problem with vitamin D RCTs is that they generally give one vitamin D dose to those in the treatment group. When it is 2000 IU/d, it increases 25(OH)D concentration by about 12 ng/mL in the US [60]. Thus, it permits examination of health benefits over a small range of 25(OH)D concentrations. Alternatively, when the dose is 100,000 IU/month of vitamin D, the equivalent of ~3333 IU/day, serum 25(OH)D can rise rapidly at first, then decline during the month, resulting in a variable 25(OH)D concentration (fluctuation of D3 and 25(OH)D concentrations). The characteristics of the participants can also have a large impact on the outcomes. For example, the age of the participants, which affects the likelihood of disease risk. For many diseases, risk increases at an increasing rate above the age of 60 years. Another factor is BMI/weight and visceral adiposity. For example, in VITAL study [60], those in the treatment arm with BMI < 25 kg/m^2^ had a significant 25% reduced risk of all-cancer incidence, while those with higher BMI did not have significant reductions, even though the mean 25(OH)D in those three categories had similar increases of 12 or 13 n/mL in serum 25(OH)D concentration.

### 3.2. Observational Studies

There are numerous observational studies that show associations between solar UVB doses and/or serum 25(OH)D concentrations and health outcomes [2,83]. Many of them report significant benefits for higher UVB doses or serum 25(OH)D concentrations. There is the stigma that “association does not equal causation” [43]. That may be true for pharmaceutical agents and for any single observational study. However, once there are several observational studies with similar results―establishing the trend- that can form the basis for analysis of causality using Hill’s criteria for causality in a biological system [3].

It is noted that well-conducted observational studies generally find results like those found in well-conducted RCTs [84]. This finding is supported by a pair of reviews published in the New England Journal of Medicine in 2000. The first one used 136 reports (53 observational studies and 83 RCTs) to compare 19 diverse treatments [84]. Seven treatments were examined for cardiologic treatments. Only two of those treatments had the combined magnitude of the effect from observational studies lie outside the 95% confidence interval for the combined effect in the RCTs. This review provides good evidence that properly conducted RCTs and observational studies can have substantial agreement in findings. The second review examined 99 reports for five clinical topics [85]. It concluded, “The results of well-designed observational studies (with either a cohort or a case–control design) do not systematically overestimate the magnitude of the effects of treatment as compared with those in randomized, controlled trials on the same topic.”

A Cochrane Methodology Review Group meta-epidemiology study regarding the comparison of observational study designs compared with RCTs was published in 2022 [86]. It included 34 reviews. The relative effect was 1.08 (95% CI, 1.01–1.18). Some discrepancies were found among the studies included, due to substantial heterogeneity among the studies suggesting bias, as well as with observational studies with unclear methods or insufficient information about study design.

A 2025 review evaluated agreement between individual nutrition RCTs and cohort studies [87]. It included 64 RCT/cohort study pairs covering over 4.1 million participants. “Regarding [population, intervention/exposure, comparator, and outcome] similarity, 20.3% pairs were “more or less identical”, 71.9% “similar but not identical” and 7.8% “broadly similar”. “Effect estimates across RCTs and cohort studies were in high agreement (relative risk [RR] = 1.00 (95% CI, 0.91–1.10, *n* = 54).” While not discussed in that review, one reason why results from cohort studies may differ from RCTs is that when variables are measured only at baseline, “regression dilution” increases with increasing follow-up periods [88]. This problem is discussed later in this review.

As discussed above, the authors suggest that the explanation for why results from vitamin D RCTs differ so greatly from results from observational studies is that they do not include participants with similar characteristics regarding vitamin D status. Those who are most likely to benefit from vitamin D supplementation are those with VDD, while those most likely to participate in vitamin D RCTs do not have VDD.

Observational studies related to vitamin D useful for causality evaluations include geographical and temporal ecological studies related to solar UVB doses, PCS, and nested case-control studies related to serum 25(OH)D concentrations, vitamin D intake, or personal UVB exposure. Cross-sectional studies are less often used regarding causality since it can be difficult to show that exposure appeared prior to health. The advantages of observational studies include the large number of participants possible, as well as large ranges of the vitamin D index. Since there are existing databases such as the National Health and Nutrition Examination Survey (NHANES) in the US and the UK Biobank, many retrospective analyses of large observational studies can be conducted quickly and inexpensively. These databases also include individual measurements of health-related variables, thereby permitting adjustments for confounding factors.

Observational studies have provided the bulk of the epidemiological findings regarding nutrients, especially vitamin D; solar UVB exposure and serum 25(OH)D concentrations on health outcomes. For example, the first suggestion that vitamin D could reduce the risk of cancer came from a geographical ecological study of annual solar radiation and colon cancer mortality rates in the US published in 1980 [89]. That was quickly followed by PCS of dietary vitamin D and calcium intake and incidence of colon cancer [90] and serum 25(OH)D concentrations and incidence of colorectal cancer [31]. In 2002, an ecological study identified 13 types of cancer in the US that had significant inverse correlations between solar UVB doses and mortality rates [91].

**Figure 1:**
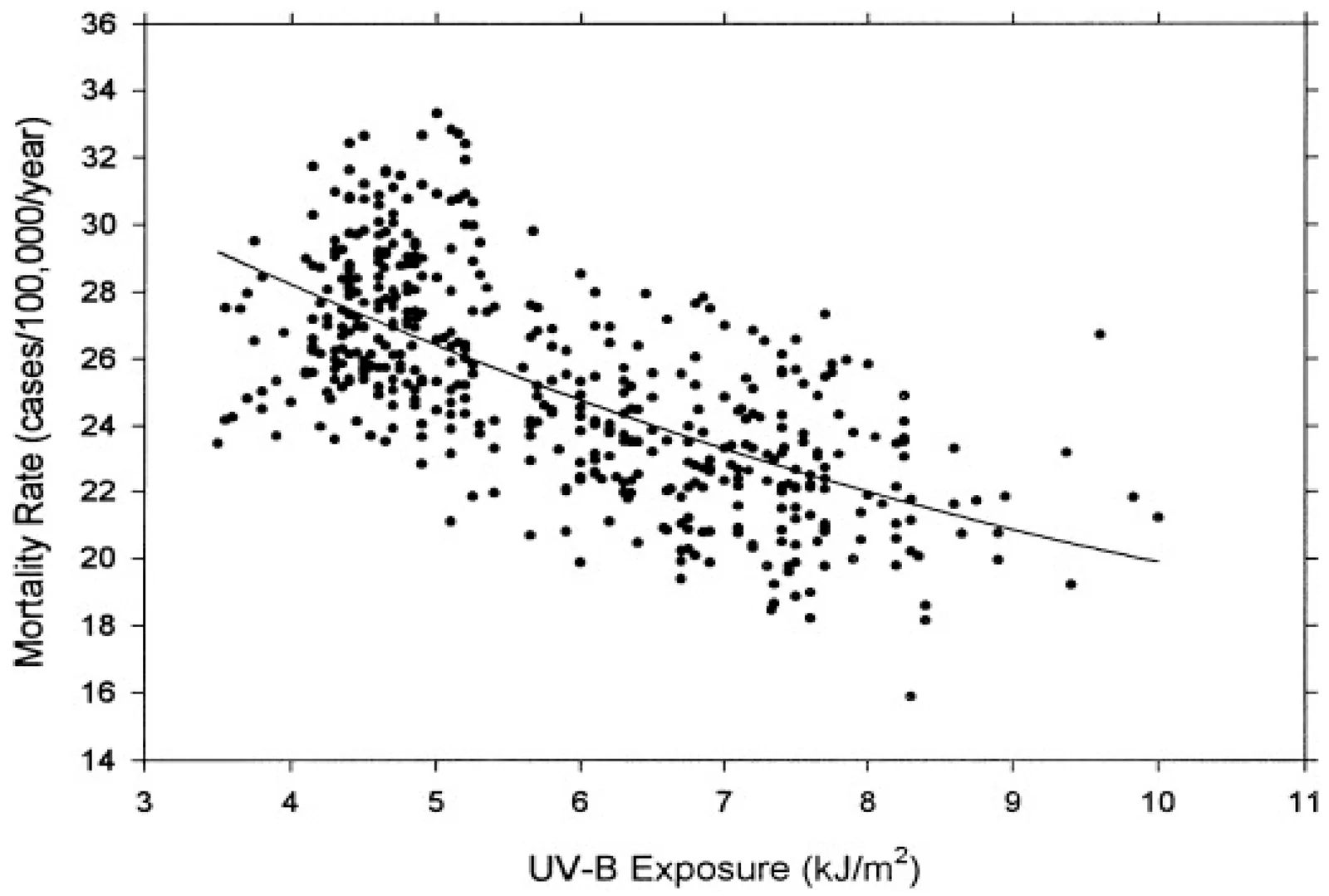
Annual mortality rates for breast carcinoma in white females (1970–1994) versus DNA-weighted ultraviolet (UV) radiation for July 1992 from the Total Ozone Mapping Spectrometer (TOMS) [91]. Reproduced by License Number 633720731908 dated 27 August 2026 from John Wiley and Sons as published in Cancer.

Figure 1 is a scatter plot of breast cancer mortality rates for white females for the period 1970–1994 [92] vs. solar UVB doses at the surface in 1992 from the Total Ozone Mapping Spectrometer [93]. Both were for ~500 state economic areas. During that period, Americans were still spending reasonable amounts of time outdoors, and a few were obese. Also, it was not well understood how to prevent and treat cancer. Thus, these results are a good representation of the natural effect of vitamin D on cancer mortality rates. Figure 1 illustrates the relationship between the UV-B exposure during 1970–1994 and the mortality rates for breast cancer.

That study was updated by adding two types of cancer and various confounding factors, including alcohol consumption, Hispanic heritage, lung cancer (assumed to be a proxy for smoking), poverty, and urban vs. rural residence [94]. Importantly, the statistical relationship between solar UVB and cancer mortality rate changed very little in the multivariable analysis.

Another ecological study of cancer was also reported in 2006. Over three million incident cancer cases between 1998 and 2002 and three million cancer deaths between 1993 and 2002 in the continental US were regressed against daily satellite-measured solar UVB doses [95]. In general, mortality rates had higher correlations with solar UVB than cancer incidences. For example, the RR of colon cancer for males for low vs. high doses was 1.11 (95% CI, 1.08–1.13) for incidence and 1.27 (95%, 1.24–1.0) for mortality.

An ecological study of U.S. cancer incidence (2016–2020) examined associations with solar UVB exposure, lung cancer, diabetes, and obesity [94]. For several cancers, including breast, colorectal, and renal cancers, obesity showed stronger associations than solar UVB exposure. Similar findings were reported in the UK using the visceral adiposity index for these and other cancers [96]. Together with the findings of the VITAL trial [60], these studies suggest that visceral adiposity and obesity may attenuate (or negate) the protective effects of vitamin D against cancer. This concept was examined in detail in a recent review [8], building on findings from an earlier review [97] which concluded that excess adipose tissue promotes chronic inflammation through mechanisms that vitamin D cannot fully counteract.

The first large prospective study to evaluate cancer incidence and mortality in relation to vitamin D was conducted by researchers at Harvard in 2006 [98]. Using data from the Health Professionals Follow-up Study, investigators developed a validated “vitamin D exposure” index based on dietary and supplemental vitamin D intake, skin pigmentation, adiposity, geographic location, and leisure-time physical activity. The model, initially derived from 1095 men, was applied to the full cohort of 47,800 participants. During follow-up, 4286 incidences of cancers and 2025 cancer deaths were recorded. Each estimated 10 ng/mL increase in serum 25(OH)D was associated with a lower incidence by 2–70% of 11 cancer types, with statistically significant reductions for colorectal, esophageal, oral/pharyngeal, and pancreatic cancers, and leukemia. The adjusted odds ratio (aOR) for overall cancer incidence per 10 ng/mL increase in 25(OH)D was 0.83 (95% CI, 0.74–0.92), while the corresponding aOR for cancer mortality was 0.71 (95% CI, 0.60–0.83).

Temporal ecological studies have also been very useful. Scragg pointed out in 1981 that the seasonality of CVD, with highest rates in winter, was likely due to seasonal variations of solar UVB and serum 25(OH)D concentrations [99]. Ironically, after publishing a paper in 2017 arguing that, due to limitations of vitamin D supplementation trials, observational studies will continue to help determine the role of vitamin D in health [100], he reversed his opinion due to the publication of a meta-analysis of 14 RCTs finding no reduction in CVD rates from vitamin D [101,102].

The first PCS of CVD risk with respect to serum 25(OH)D concentration was conducted on 1739 participants of the Framingham Offspring Study [103]. During a mean follow-up of 5.4 years, 120 individuals experienced the first CVD event. The adjusted hazard rate (aHR) for <15 ng/mL vs. >15 ng/mL was 1.62 (95% CI, 1.11–2.36). When separated by hypertension status, the aHR for those with hypertension was 2.13 (95% CI, 1.30–3.48), while for those without hypertension, the aHR = 1.04 (95% CI, 0.55–1.96).

**Figure 2:**
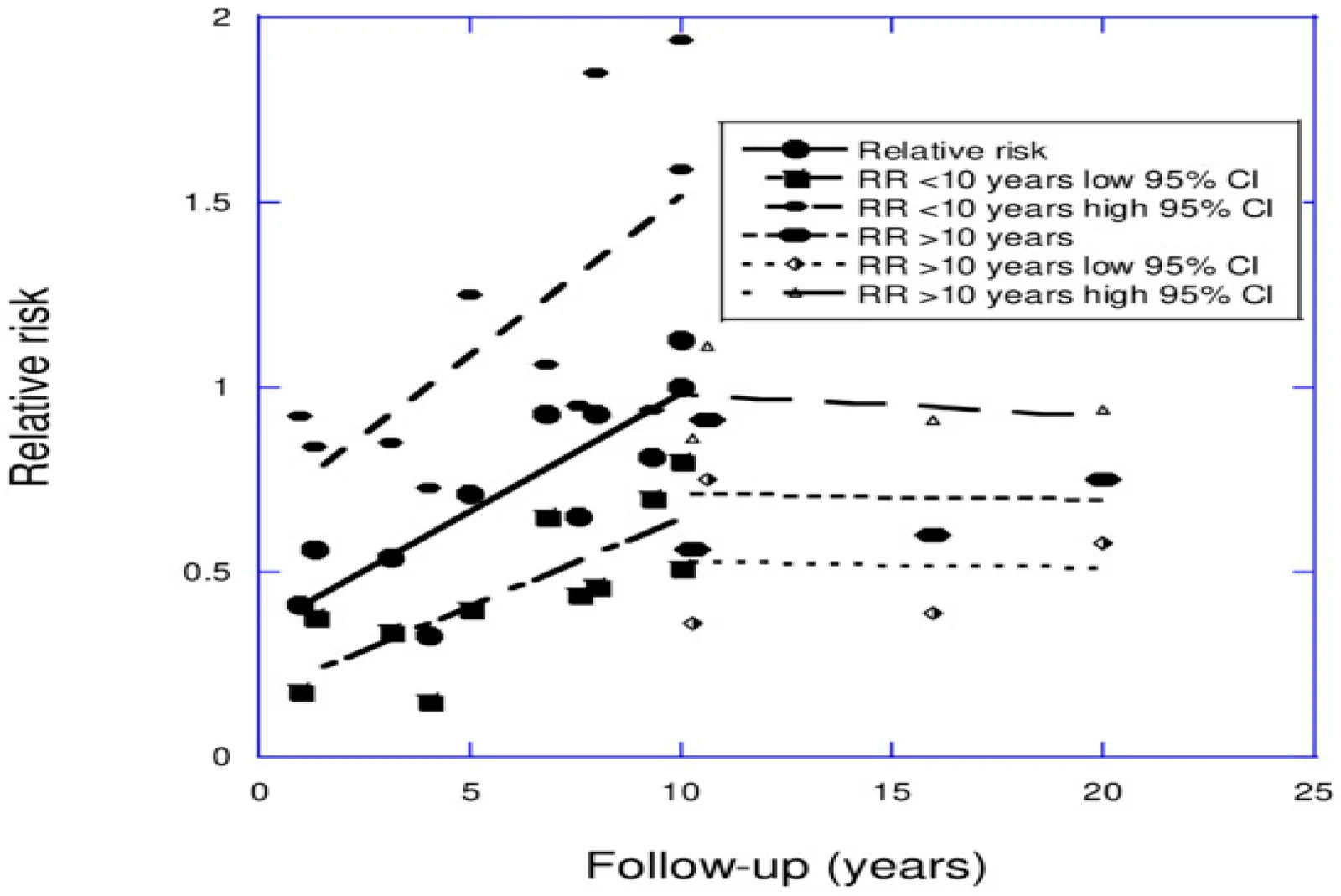
Plot of RR of stroke versus years of follow-up concerning high vs. low 25(OH)D concentrations (mostly >30 vs. <20 ng/mL) from PCS [106]. This figure is from an open-access article distributed under the terms and conditions of the Creative Commons Attribution (CC BY) license (https://creativecommons.org/licenses/by/4.0/, accessed on 10 December 2024).

The observational study evidence that vitamin D reduces the risk of CVD is very strong [83,104,105,106]. The evidence from RCTs is weak because the trials were not based on participants with VDD. Figure 2 shows the effect of regression dilution on the RR of stroke for high vs. low 25(OH)D concentrations.

There are enough reported observational studies to carry out a well-designed meta-analysis on the effects of vitamin D on cancer incidence and mortality [107]. For PCS, the results are usually analyzed using proportional hazards analysis and forest plots in which the weight for each study is based on the number of participants. For PCS, the results are usually analyzed using proportional hazards analysis and forest plots in which the weight for each study is based on the number of participants. However, this approach overlooks an important limitation of PCS, “the above-mentioned regression dilution,” due to changes in the values of the variables over time. This limitation was first reported in 1999 by Clarke and colleagues [88]. They plotted systolic BP, diastolic BP, and blood cholesterol measured every two years for 26 years for participants in the Framingham Study. The plots showed that the spread between the highest and lowest quintiles determined at baseline decreased by 40–50% by the tenth year and by 60–70% by 26 years. Thus, shorter follow-up periods will have HRs, ORs, or RRs with lower regression dilutions but will have larger 95% CI values due to smaller numbers of cases, thus reducing the statistical validity at extreme durations.

This regression dilution effect has been demonstrated for PCS of health outcomes related to serum 25(OH)D concentrations for colorectal cancer, stroke and major cardiovascular events (MACEs), and dementia, Alzheimer’s disease (AD) and cognitive impairment, reviewed by Grant and colleagues [2]. The general finding was that the apparent risk decreases in a linear manner to near zero after about ten years, after which it becomes more varied (see Figure 2). There is also another factor involved, namely the age of the participants and the change in risk of the health outcome of interest with age. This effect can counter the effect of regression dilution in some cases. 

### 3.3. Observational Studies Are Better than RCTs for Evaluating Nutrients, like Vitamin D

Many non-drug therapies, particularly micronutrients such as vitamin D, do not align with the RCT framework. Micronutrients differ fundamentally from synthetic pharmaceuticals in dose–response behavior and biological roles, making RCTs an unsuitable method for evaluating their effects. These interventions are more appropriately assessed through large, longitudinal, community-based cohort studies.

Micronutrient therapies are often individualized, dependent on practitioner expertise and patient interaction, and difficult to blind effectively. Their effects may be rapid, broad, and holistic, whereas RCTs are designed to detect narrow, isolated outcomes in uniform populations. Moreover, these therapies frequently rely on integrative diagnostic approaches and outcomes—such as vitality, well-being, and disease prevention—that are not easily quantified within conventional RCT structures. Pharmaceuticals, by contrast, are engineered to target specific signs and symptoms, and their regulatory approval is based on RCT evidence. As a result, effective micronutrient therapies, including vitamin D, are often marginalized not due to lack of benefit but because they do not conform to the “one drug, one disease” model underlying RCT methodology.

Placebo effects (approximately 30%) and investigator or observer bias can exaggerate perceived treatment efficacy, particularly for subjective outcomes such as pain or mood. These biases are less relevant for objective biological endpoints such as HbA1c reduction, cardiac ejection fraction, or tumor size. Although RCTs can mitigate such biases, they are not universally required. Furthermore, large RCTs demanded by regulators are prohibitively expensive, limiting participation to major pharmaceutical companies, large foundations, or government agencies capable of funding extensive trials, such as VITAL [60].

Following the 1962 legislation intended to prevent another thalidomide-like event, the FDA interpreted “well-controlled investigations” to require large, randomized double-blind trials for drug approval. The substantial financial and institutional investment in this system has entrenched the RCT model, leading many stakeholders to defend it and resist alternative evidence frameworks. Consequently, researchers often avoid topics that challenge prevailing paradigms, as contradictory findings are difficult to publish and may jeopardize grant funding.

A 2014 Cochrane Review demonstrated that small, unblinded observational studies frequently yield results comparable to large RCTs, likely because such studies are conducted only when effect sizes are clinically obvious [108]. This is important because observational studies are inexpensive, replicable, and accessible, offering a means to challenge the pharmaceutical industry’s dominance over definitions of medical evidence. This study demonstrated significant inverse associations between serum 25(OH)D concentrations and the incidence and mortality of multiple diseases. These findings align with numerous observational studies linking vitamin D deficiency to increased disease risk and, for several conditions, satisfy multiple Hill’s criteria supporting causality. Therefore, for many diseases discussed, routinely concluding that “additional studies are needed to establish causality” is no longer warranted. Future research should instead focus on defining optimal 25(OH)D thresholds, identifying responsive subpopulations, and developing effective implementation strategies to improve health outcomes.

### 3.4. Hill’s Criteria for Causality in a Biological System

Given the fact that most vitamin D RCTs were not designed to evaluate causality for vitamin D in reducing risk of disease incidence, progression, and/or mortality, it is necessary to investigate alternative approaches for causality for vitamin D. A.B. Hill proposed a set of criteria for evaluating causality in a biological system in his presidential address to the Royal Society of Medicine in 1965 [3]. As of 22 June 2026, this paper was cited by 8626 publications according to SCOPUS and by 14,097 publications according to Google Scholar. The criteria are given in Table 3. To establish causality, it is only necessary to satisfy some of Hill’s criteria, not all of them [3].

The more criteria are satisfied, the stronger the case for causality. In several respects, satisfying Hill’s criteria represents stronger evidence for causality than does finding a statistically significant better outcome in an RCT. The primary reason is that a much greater body of evidence is used rather than just the result of supplementing a selected group of people with a single vitamin D dose. Table 3 lists Hill’s criteria for causality important for vitamin D. Not all need to be satisfied to claim causality, but the more that are, the better.

There have been approximately 20 Hill’s criteria analyses in the peer-reviewed journal literature. Those that provide good analyses at the time of publication are reviewed here. Such analyses are reviewed here for nine diseases: cancer, CVD, hypertension/high BP, COVID-19, T1DM, T2DM, periodontal disease, multiple sclerosis, and dementia.

#### 3.4.1. Cancer

Risk of cancer due to low solar UVB/vitamin D has been reported since 1980 [89]. Thus, by 2009, there had been numerous geographical ecological studies of solar UVB and cancer, as well as nested case-control studies of serum 25(OH)D concentration and cancer incidence, to do a Hill’s criteria analysis regarding vitamin D and cancer, as done by Grant [110]. At that time, geographical ecological studies and a few PCS had identified 16 types of cancer for which higher solar UVB and/or serum 25(OH)D concentration was associated with reduced risk of cancer incidence and/or mortality rates. The types of cancer exhibiting the strongest effects of UVB and/or vitamin D were breast and colorectal cancer [107].

As shown in Figure 1 in this review, the regression fit to breast cancer mortality rates vs. solar UVB doses fell by 30% going from lowest to highest UVB doses. Most of the ecological study and PCS findings for the 16 types of cancer were similar across numerous studies in the US, Europe, and Asia. Temporality is satisfying for ecological studies since it can take many years to progress from initiation to clinical detection. For example, sigmoidoscopy was recommended every ten years and was found to significantly reduce the risk of cancer of the rectum and distal colon [111]. As for dose–response relationships, a meta-analysis of five nested case-control studies of CRC incidence with respect to pre-diagnosis serum 25(OH)D concentration found a 50% lower risk of CRC for serum 25(OH)D concentration > 33 ng/mL vs. <12 ng/mL [112].

As for plausibility, the mechanisms well known at that time included improved differentiation and apoptosis [113] to reduce incidence, plus antiangiogenesis [114] and anti-metastasis [115] to reduce progression and mortality. There has been one RCT regarding vitamin D supplementation and risk of cancer [116]. The vitamin D dose was 1100 IU/day of vitamin D_3_, and in one arm, 1400–1500 mg/day of supplemental calcium/day. Those taking both vitamin D_3_ and calcium had a 77% reduction in all-cancer incidence, of which 35% was attributed to vitamin D_3_. As for analogy, the ecological studies of solar UVB and cancer were considered analogous to the effects of serum 25(OH)D concentrations [107]. On the other hand, beta-carotene was thought to reduce the risk of lung cancer based on observational studies, but beta-carotene supplementation studies found it increased the risk of lung cancer [117]. It was realized later that beta-carotene is only one component of fruits and vegetables.

In 2012, Mohr and colleagues evaluated Hill’s criteria for the evidence that vitamin D reduces risk of breast cancer [118]. Strength of association for serum 25(OH)D concentration was evaluated based on a meta-analysis of observational studies [119]. There was a 47% lower risk of breast cancer for high vs. low 25(OH)D concentrations based on case-control studies and 13% based on nested case-control studies. The results for nested case-control studies were thought to be lower than actual due to changes in serum 25(OH)D concentrations during long follow-up periods. Consistency was judged to be strong based on similar results from many types of studies, including latitudinal studies, case-control and nested case-control studies of serum 25(OH)D concentrations, laboratory studies, oral vitamin D studies, and RCTs. Temporality was demonstrated in nested case-control studies and the RCT.

Biological plausibility was claimed through reference to laboratory studies, such as prevention of loss of E-cadherin, which binds epithelial cells together to keep cells in a well-differentiated state [120]. They also discussed the Disjunction-Initiation-Natural selection-Overgrowth-Metastasis-Involution-Transition (DINOMIT) model [121]. As for confounding factors, they suggested that none of the ones considered could account for all the differences in breast cancer incidence between individual women. Although taken together, the factors could make a substantial contribution to breast cancer incidence at the population level. However, given the ubiquitous nature of vitamin D in populations, it is expected to account for a greater proportion of excess risk for breast cancer than factors of lower prevalence [107]. Jumping ahead, it was found in an ecological study of cancer incidence in the US that obesity competes with solar UVB for explaining breast cancer and CRC in the period 2016–2020 due to the high obesity rates [94].

In 2020, Sluyter, Manson, and Scragg used Hill’s criteria to analyze vitamin D and clinical cancer outcomes [122]. They noted that case-control studies are susceptible to reverse causality, changes in lifestyle after cancer diagnosis, etc., while that is not the case for prospective studies, which they emphasized. They also noted that vitamin D does not fulfill one Hill criterion, specifically, since vitamin D affects many diseases. They concluded, “Observational studies showed that, in many cases, low vitamin D was inversely associated with cancer outcomes [107]. For this, the associations for some outcomes, particularly colorectal cancer, seem to fulfill some (but not all) of Bradford-Hill’s criteria for causation, including consistency of findings across different meta-analyses and primary studies, temporality (prospective study associations), biological gradient (dose–response associations), and strength of associations (strong in some cases).”

In 2022, Muñoz and Grant published a comprehensive review of vitamin D and cancer [123]. It included tables with results from geographical ecological studies, meta-analyses of cancer incidences with respect to serum 25(OH)D concentrations, an analysis of the effect of regression dilution in PCS, and many findings regarding the mechanisms whereby vitamin D reduces risk of cancer [107]. There were four ecological studies regarding cancer incidence and five regarding cancer mortality. Twenty-four types of cancer had incidence inversely related to solar UVB in at least one study, while 25 types of cancer did for mortality [107].

For CRC, it was shown that by plotting RR for high vs. low 25(OH)D concentration vs. years of follow-up in PCS, the extrapolation of the linear fits to the data for men and women each at zero follow-up time was near 0.75. Thus, this analysis of the effect of regression dilution showed that all the PCS consistently found a reduced risk of CRC, with the RR affected by follow-up time. A few RCTs have been performed, finding modest effects for vitamin D supplementation [124]. A recent RCT from Japan found that vitamin D supplementation had a very strong impact on survival from gastrointestinal tract cancers for patients with p53 antibodies [32].

#### 3.4.2. Cardiovascular Disease

The role of solar UVB and vitamin D in affecting risk of CVD was proposed in 1981 [99]. Grant and Boucher published an analysis of the regression dilution effect on risk of MACE and stroke in 2024, in which they assessed the causal links between vitamin D and CVD using Hill’s criteria [106]. The plots of RR for high vs. low 25(OH)D concentration for stroke and low vs. high 25(OH)D concentration for MACE found that the regression fits to the data showed the shortest follow-up periods were associated with reduced risk of stroke by about 60% and increased risk of MACE by about a factor of two. These findings satisfied several of Hill’s criteria: strength of association, consistency, temporality, and biological gradient. Fourteen mechanisms were listed regarding how vitamin D reduces risk of CVD. Some of these were antifibrotic, antihypertrophic signaling [125], endothelial function maintenance [126], insulin resistance risk reduction [127], MMP-2 and MMP-9 activity reduced [128,129], and reduction in arterial pressure through effects on endothelial and muscle cells [130].

RCTs published by 2024 have not demonstrated that vitamin D supplementation reduces the risk of stroke or CVD events. The reason suggested was that not enough participants are enrolled in VDD. That contention was supported by a recent paper [131]. that evaluated two major vitamin D RCTs, VITAL [60] and D-Health [66]. It compared the distribution of baseline 25(OH)D concentrations in those trials with serum 25(OH)D concentrations found in the UK Biobank and risk of CVD mortality. There were very few participants with vitamin D-deficient or insufficient 25(OH)D concentrations in either RCT. Using the increases in 25(OH)D concentrations reported in the two RCTs, about 12 ng/mL, the calculations for 25(OH)D concentrations and risk of CVD for individuals in the UK Biobank indicated that hazard ratios for CVD mortality would be between 0.74 and 0.79.

Confounding is a concern. CVD incidence and mortality rates are much higher in winter than in summer [132,133]. Winter is when serum 25(OH)D concentrations are lowest. As discussed in another Hill’s criteria for vitamin D and CVD [83] a large study of serum 25(OH)D concentrations measured by the Mayo Clinic in the US from 2006 to 2015 found that the percentage of values below 12 ng/mL rose from 1.5% in summer to 9% in winter, while the percentage of values from 12 to 24 ng/mL rose from 17% to 28% [78]. Cold temperatures in winter, as well as seasonal changes in gene expression, may also contribute to increased risk of CVD events in winter. However, inspection of Figure 2 in Oskarsson et al., 2025 [134] finds that the peak CVD mortality rate in Europe is in April (ignoring the January peak due to the holidays), the month with the lowest 25(OH)D concentrations, while the lowest mortality rate is in September, the month of the highest 25(OH)D concentrations. That differs from the low and high temperatures in Europe, near the low at the end of January and near the high at the end of July.

Grant and Boucher discussed the role of parathyroid hormone (PTH) in the risk of CVD [133]. “PTH stimulates aldosterone secretion by increasing calcium concentrations in the cells of the adrenal zona glomerulosa as a result of binding to the PTH/PTHP-rP receptor and indirectly by potentiating angiotensin-two-induced effects [135]. A study conducted in Utah found increased risk of CVD prevalence/incidence rates for PTH > 75 pg/mL by factors of 1.25 to 3 [136]. An analysis of PTH and 25(OH)D in >300,000 US patients found that PTH increases with age and is inversely correlated with 25(OH)D concentrations [137]. “Patients most likely to have PTH > 75 pg/mL were older than 60 years and had 25(OH)D concentration < 10 mg/mL.” “A study of seasonal variations of serum 25(OH)D concentrations in British adults aged 45 years indicated that 16% had serum 25(OH)D concentrations < 10 ng/mL in winter compared with 3% in summer [138].” Thus, the seasonal variation in CVD events can be explained in part by seasonal variations in 25(OH)D and PTH concentrations (or the combination effect).

#### 3.4.3. Hypertension/Blood Pressure

It was reported in 1976 that BP was highest in winter [139]. Both temperature and serum 25(OH)D concentrations are lowest then. An open-label vitamin D supplementation study was conducted with community-dwelling participants in Canada given vitamin D supplements and counseled on how to achieve a 25(OH)D concentration > 100 ng/mL [140]. After a year of supplementation, hypertensive patients reduced mean systolic, diastolic, and mean arterial pressure by 12–18 mmHg irrespective of whether they were taking BP medications. The authors conducted Hill’s criteria analysis for vitamin D and lowering BP to reduce risk or prevalence of hypertension. Strength of association was satisfied by the results of their vitamin D supplementation study. Consistency was supported by noting that several other studies had found that vitamin D supplementation had benefits for hypertension. Temporality was supported by their vitamin D supplementation study.

A dose–response relationship is suggested with increasing serum 25(OH)D from the deficient range to the physiological range (>100 nmol/L [40 ng/mL]) and associated with significant reductions in systolic, diastolic, and mean arterial pressure. (A meta-analysis of PCS found that the risk of hypertension was highest for 25(OH)D concentration = 6 ng/mL (RR = 1.38 (95% CI, 1.15–1.62) [141].) The mechanisms whereby vitamin D may lower BP include immunomodulatory and anti-inflammatory effects, as well as through its actions on the renin-angiotensin activity [140] as an endogenous inhibitor leading to a decrease in BP [142]. Vitamin D may also act to improve endothelial function [143] and attenuate the effect of parathyroid hormone [144]. As for confounders, they noticed a greater reduction in BP in summer than in winter, which they ascribed to possible confounding factors in winter. An obvious possibility is cold weather. A meta-analysis of PCS found that the risk of hypertension was highest for 25(OH)D concentration.

PTH also affects BP. An analysis of data from the UV National Health and Nutrition Examination Surveys (NHANES) during 2003–2013 found that PTH had a much stronger effect on BP than did 25(OH)D concentration [145]. The mean effects of PTH for the highest vs. lowest quintile were 5.9 mmHg for systolic BP and 4.5 mmHg for diastolic BP. The effects of 25(OH)D concentration for those PTH quintiles were 2.2 mmHg for systolic BP and 0.8 mmHg for diastolic BP.

#### 3.4.4. COVID-19/(SARS-CoV-2)

It was proposed early in 2020 by Grant and colleagues that vitamin D could help reduce the risk, severity, and progression of COVID-19 [146]. The evidence was based on epidemiology and mechanisms. Several RCTs found that vitamin D reduced risk of epidemic influenza. Vitamin D has also been shown to reduce risk of illness from several enveloped viruses. An ecological analysis of case-fatality rates for pandemic influenza in the US in 1917–1918 indicated that deaths due to pneumonia after developing influenza were lower in regions with higher solar UVB doses [147]. The proposed mechanisms whereby vitamin D could reduce risk of COVID-19 included the induction of human cathelicidin and defensins that could lower viral replication rate and reduce concentrations of pro-inflammatory cytokines that injure the lining of the lungs, leading to pneumonia [148,149], as well as increasing concentrations of anti-inflammatory cytokines.

Hill’s criteria analysis of vitamin D and COVID-19 was reported by Mercola and colleagues in 2020 [148]. Strength of association and dose–response were demonstrated for SARS-CoV-2 positivity [35] as well as for COVID-19 [150]. Consistency was supported by 13 observational studies reporting associations between serum 25(OH)D concentration and risk of COVID-19 incidence, severity, and mortality by 27 September 2020 according to PubMed.gov. The mechanisms identified included those discussed above, as the well as activation of the innate immune system, increasing angiotensin-converting enzyme 2 (ACE2) concentrations [6,151], and reducing the risk of death from ensuing acute respiratory tract syndrome, reducing the risk of endothelial dysfunction [152], reducing MMP-9 concentrations [128], and reducing risk of the bradykinin storm [153]. Vitamin D also helps to strengthen the adaptive immune system. As for experimental verification, there were two weak vitamin D supplementation studies on COVID-19 patients [154,155].

Wimalawansa provided additional Hill’s criteria analysis for SARS-CoV-2/COVID-19 in 2025 [149]. As it was published several years after the peak of the pandemic had passed, it had a greater set of evidence to incorporate. For example, there were multiple meta-analyses and trial sequential analyses to confirm the role of vitamin D supplementation in reducing disease incidence, complications, and mortality. One systematic review was reported involving one RCT and four non-randomized studies of intervention (NRISs) for primary prevention and two RCTs and three NRISs for secondary prevention [156,156]. While the analysis found no significant effect on risk of COVID-19, it found a reduced risk of ICU admission (RR = 0.35 [95% CI, 0.20–0.62]) and mortality (RR = 0.46 [95% CI, 0.30–0.70]). Another review including two RCTs and 27 cohort studies found reduced risk of severe COVID-19 (RR = 0.38 [95% CI, 0.20–72] in 6 studies) and mortality (RR = 0.35 [95% CI, 0.17–0.70] in 8 studies) [157].

#### 3.4.5. Diabetes Mellitus

Hill’s criteria analyses for both T1DM and T2DM were reported in 2017 by Altieri and colleagues [158]. For T1DM, a meta-analysis of eight studies (two PCS and six case-control studies) on vitamin D intake during early life was reported in 2013 [159]. The pooled odds ratio for T1DM comparing vitamin D supplementation with non-supplementation during early life was OR = 0.71 (95% CI, 0.51–0.98). This meta-analysis was cited as satisfying strength of association, biological gradient, and consistency. The strongest evidence for vitamin D supplementation reducing risk of T1DM was from an observational study of incidence in Finland.

As reported in 2010 by Hypponen et al. [160], over a 30-year period, those who took 2000 IU/d vitamin D during the first year of life had an RR = 0.22 (95% CI, 0.05–0.85) of developing T1DM compared to those who took lower doses. This study was used to satisfy temporality. As vitamin D supplementation for infants in Finland decreased, T1DM incidence rates increased. There was a nearly linear increase in incidence rates for those under the age of 15 years from 1965 to 1992, after which the rate of increase nearly doubled; vitamin D supplementation for infants was 2000 IU/d in 1964, dropping to 1000 IU/d in 1975 and 400 IU/d in 1992 [161].

Plausibility: Vitamin D has been shown to have effects on the immune system related to T1DM, as well as a slow decline of residual β-cell function [162]. Vitamin D regulates components of the immune system and regulates calcium homeostasis, which is important for immune function and insulin secretion [163]. Experiment: Supplementation of those with new-onset T1DM with 2000 IU/d vitamin D_3_ used as adjunctive therapy with insulin was associated with a protective immunologic effect (increased chemokine ligand 2 and regulatory T cells and reduced production of stimulated C-peptide) and slower decline of residual β-cell function [162]. Analogy: T1DM often occurs in those with multiple sclerosis, which is linked to vitamin D deficiency [164]. Thus, Hill’s criteria for causality are reasonably well satisfied for vitamin D in reducing the risk of T1DM. However, clinical trials finding that vitamin D supplementation reduces risk of T1DM appear to be lacking.

For T2DM, Strength of association: The RR for incidence of T2DM from 21 prospective studies, high vs. low 25(OH)D quantile, is 0.62 (0.54–0.70) [165]. Consistency: Out of 21 PCS, the random-effects meta-analysis found an RR of T1DM incidence for high vs. low 25(OH)D = 0.62 (95% CI, 0.54–70) [165]. Temporality: PCS found an inverse correlation between serum 25(OH)D concentration and incidence of T2DM [165]. Biological gradient: The meta-analysis of incidence of T2DM with respect to 25(OH)D concentration in PCS showed RR increased from ~0.75 at 30 ng/mL to 1.00 at ~14 ng/mL [165]. Plausibility: The mechanisms by which vitamin D might reduce the risk of T2DM were reviewed by Mitri et al. [166]. They include supporting insulin secretion: the “majority of observational studies [find] that vitamin D is positively correlated with insulin sensitivity, and its role is mediated both by direct mechanisms through the availability of vitamin D receptors in several tissues and indirectly through changes in calcium levels [167].

Experiment: Clinical trials for vitamin D supplementation in reducing the incidence of T2DM or having any impact on glucose control and insulin resistance in general populations [167]. However, a clinical trial in Iran, where vitamin D deficiency is common, has shown that vitamin D plus calcium supplementation can decrease fasting glucose [168]. The baseline 25(OH) D concentration was 38 nmol/L (15 ng/mL). The successful result was most likely due to the low baseline concentration. Analogy: Health outcomes with similar findings for the criteria include CVD [169]. Confounding factors: Obesity is an important risk factor for T2DM; those with high body mass index generally have low 25(OH)D concentrations. While studies of 25(OHD and incidence of T2DM try to correct for body mass index, it is possible that it is not fully corrected for [170].”

The evidence that vitamin D reduces risk of T2DM was boosted in 2020 by the reanalysis of results from an RCT involving progression from prediabetes to diabetes [171]. When the analysis was based in achieved serum 25(OH)D concentration for those treated with 4000 IU/day vitamin D_3_ for 2.5 years, it was found that those who achieved >50 ng/mL had a RR = 0.29 (95% CI, 0.17–0.50), and that each 10 ng/mL increase was associated with RR = 0.75 (95% CI, 0.68–0.82).

#### 3.4.6. Periodontal Disease

Hill’s criteria were evaluated for periodontal disease (PD) in 2010 by Grant and Boucher [172]. Strength of association. A study of participants in NHANES in 1988 and 1994 was reported in 2004 [173]. It found that for participants > 50 years of age, periodontal attachment loss for 25(OH)D concentration < 40 nmol/L vs. >85 nmol/L for men had β coefficients = 0.58 (95% CI, 0.40–0.75) adjusted for age and race or ethnicity and 0.39 (95% CI, 0.17–0.60) when adjusted for many risk-modifying factors. For women, the values were 0.46 (95% CI, 0.30–0.62) and 0.26 (95% CI, 0.09–0.43), respectively. Trends with respect to serum 25(OH)D concentrations were significant at *p* = 0.001. In a subsequent study, Dietrich found a significant trend for gingival bleeding across quintiles of 25(OH)D concentration [174] Consistency was suggested to be satisfied by several studies reporting African Americans having higher rates of PD than white Americans. Temporality. There were no PCS by 2010. The two Dietrich et al. papers were based on cross-sectional studies, which do not examine temporality. It was suggested that nearly all the vitamin D studies using NHANES data by 2010 have been supported by other studies so that temporality could be assumed.

Mechanisms. One mechanism is that vitamin D reduces concentrations of MMPs [128], which are involved in dissolving collagen and, thus, leading to attachment loss. Another mechanism is an increase in human cathelicidin and defensin concentrations, which can reduce the viability of PD-causing bacteria [175]. Experiment. During a small 3-year RCT, 11 of the 82 subjects (13%) taking vitamin D and calcium supplements and 17 of the 63 subjects (27%) taking placebo lost one or more teeth (OR = 0.4; 95% CI: 0.2 to 0.9) [176].

However, it was not possible to separate the effects of vitamin D and calcium. Analogy. Since vitamin D reduces the risk of dental caries, which is also linked to bacteria, studies of sun exposure and risk of dental caries provide a good analogy. Two ecological studies in the US of sun exposure found a higher risk of dental caries for low exposures [177,178]. Confounding factors. While smoking, diet, and calcium intake are also risk-modifying factors for PD, when they are included in studies of PD and systemic and infectious diseases, there is often a large portion of causation unaccounted for. Bone mineral density (BMD) is also linked to calcium, diet, and smoking. A study in Pennsylvania found that BMD and changes in BMD were similar in dentate and edentulous women [179]. In addition, there was no difference in BMD for women with or without PD.

#### 3.4.7. Multiple Sclerosis

Hanwell and Banwell published Hill’s criteria analysis of vitamin D and MS in 2011 [180]. Strength of association. A case-control study from Tasmania found that high sun exposure in childhood was related to decreased risk of MS [181]. Another finding was from a case–control study nested within a prospective cohort of over 7 million US military personnel [28]. A decreased risk of MS (OR 0.38 [95% CI 0.19–0.75]) was observed among white participants (148 cases, 296 controls) with serum 25(OH)D concentrations in the highest quintile (99–153 nmol/L) compared with the lowest quintile (<63 nmol/L).

Consistency. Low vitamin D status in people with MS was reported in one study from Australia, five from the US, and four from Europe. Temporality. The US military personnel study supports temporality [28].

Biological gradient. The US military personnel study found an OR = 0.59 (95% CI, 0.36–0.97) for risk of MS for a 20 ng/mL increase in serum 25(OH)concentration [28]. Plausibility. One of the strongest mechanistic links between vitamin D and MS comes from a recent study demonstrating that calcitriol modulates the expression of the particular HLA-DRB1 allele most consistently associated with increased risk of MS, HLA-DRB1*1501 [182]. In addition, vitamin D reduces inflammation through effects on cytokines [183]. It also reduces MMP concentrations, as previously discussed, which increases the blood-brain barrier to auto-reactive immune cells [184].

Coherence. Cigarette smoking is a risk factor for MS [185]. Smoking lowers serum 25(OH)D concentrations [186]. Epstein-Barr (EB) virus is a risk factor for MS [187]. A hypothesis paper in 2007 suggested that vitamin D status modulates the immune response to Epstein-Barr virus [188]. Experiment. A limited number of small studies have explored vitamin D—and even calcitriol—supplementation in adults with established MS; such studies primarily demonstrate the safety profile of vitamin D supplementation and provide a preliminary view into efficacy. Analogy. Vitamin D is a common putative factor in numerous immune-mediated inflammatory diseases.

In the past few years, the group of Cicero Coimbra in Brazil has been using very high vitamin D_3_ doses (35,000 ± 22,000 IU/day) to treat MS patients. As reported in 2022, the safety data in terms of biological parameters, such as serum calcium, were in the normal ranges [189]. This approach can only be done with proper medical supervision. Recently, it was determined that CD4^+^ T cells activated by EB cause inflammation and nerve damage in MS [190]. Through a chain of steps, 1,25(OH)_2_D can reduce antigen-presenting capacity CD4+ T cells [191].

#### 3.4.8. Dementia

Annweiler published Hill’s criteria analysis for dementia prevention by vitamin D in 2016 [192]. Strength of association. A meta-analysis reported a 2.4-times greater risk of cognitive impairment in the case of hypovitaminosis D compared to those with adequate vitamin D status [193]. Consistency. It has been reported that AD patients exhibit lower serum 25(OH)D concentrations than do control individuals without AD [194]. Temporality. PCS shows that low 25(OH)D concentration is associated with risk of AD and all-cause dementia [195]. Coherence with known science. Vitamin D status is a biological determinant associated with brain structure. A meta-analysis showed that individuals with hypovitaminosis D exhibit cerebral lateral ventricles 1.01 standard deviations larger (a proxy for brain atrophy) than those without hypovitaminosis D [196].

Vitamin D status is also associated with cognitive function in older adults [193]. Biological gradient. In most previous studies, increased 25(OH)D concentration was associated with better cognitive performance and decreased incidence of dementia. Plausibility. Vitamin D regulates the synthesis of neurotrophic agents, such as nerve growth factor [197] and glial cell line-derived neurotrophic factor [198]. 1,25-(OH)_2_D triggers an anti-inflammatory response in brain pericytes [199]. Vitamin D attenuates the accumulation of amyloid-β 42 through phagocytosis [200]. Plausibility. The mechanisms whereby vitamin D reduces risk of AD were reviewed in 2013 [201].

An analysis of the effect of regression dilution on PCS regarding risk of AD and dementia found that the RR for low vs. high 25(OH)D concentration increased linearly from near 1.0 (no effect) for follow-up periods of 13–14 years to ~2.1 for a follow-up period of 5.5 years [202]. Experiment. A 7-year PCS involving 498 community-dwelling women (mean age 80 ± 4 years) not taking vitamin D supplements was analyzed to determine the effect of dietary vitamin D intake and other factors on risk of AD [203]. Dietary vitamin D intake had the highest reduction (OR = 0.23 [95% CI, 0.08–0.67, *p* = 0.009]) followed by midday sun exposure (OR = 0.45 [95% CI, 0.24–0.85]).

## 4. Optimal Vitamin D Supplementation and Target Serum Concentrations

Vitamin D functions very differently from pharmaceutical drugs. It acts as a “threshold nutrient”: individuals must reach adequate tissue and serum 25(OH)D levels before meaningful biological benefits appear, and additional intake beyond this point offers little added effect. It is also a “network nutrient” relying on cofactors such as magnesium, vitamin K_2_, and omega-3 fatty acids; deficiencies in these can limit vitamin D activity even when serum levels appear sufficient. Genes for vitamin D-related enzymes and their receptors are widely distributed in all human tissues, and thus, its broad clinical effects are unsurprising [4,7].

Vitamin D further serves as a “systems regulator”, influencing hundreds of genes involved in immunity, metabolism, and cellular function. Because of these nutrients’ specific characteristics, vitamin D cannot be evaluated using traditional drug-development models. Unlike pharmaceuticals with steep dose–response curves, vitamin D has a shallow, threshold-based response, making serum 25(OH)D concentration—not a fixed dose—the most meaningful indicator of sufficiency. Individuals taking the same dose may reach very different serum levels due to genetics, body composition, absorption, medications, sun exposure, pregnancy, aging, chronic illness, baseline status, and cofactor availability. Therefore, targeting an appropriate serum 25(OH)D concentration is more informative and clinically relevant than prescribing a uniform daily dose.

Recent evidence shows that different tissues require different serum 25(OH)D thresholds for activation. Essential functions—such as neuromuscular performance and stress responses—operate at lower levels, while immune regulation, metabolism, and cancer-related pathways need higher concentrations. This hierarchy limits the usefulness of defining vitamin D sufficiency at 20–40 ng/mL, as these values may support only a fraction of vitamin D–dependent functions. Maintaining serum 25(OH)D at ≥50 ng/mL, ideally 50–80 ng/mL, appears to support nearly all vitamin D-responsive processes without increasing adverse effects. Only a very small minority—those with genetic polymorphisms, cofactor deficiencies, or rare metabolic defects—require higher levels. This tissue-specific threshold model explains why a single cutoff cannot define adequacy across all systems and underscores vitamin D’s broad endocrine, immune, metabolic, and cellular roles.

Preventing infections and modulating autoimmune responses are among the strongest reasons to maintain serum 25(OH)D above 50 ng/mL. Infectious diseases and sepsis cause over 11 million deaths annually and drive major long-term health burdens. Vitamin D strengthens innate immunity, supports antimicrobial defenses, and tempers harmful inflammation, making optimal vitamin D status a cost-effective strategy to reduce infectious and autoimmune disease worldwide [13].

Contemporary research shows wide individual variability in response to the same vitamin D dose. Population guidelines help public health, but no single intake produces uniform effects because physiology, genetics, metabolism, lifestyle, body composition, and health status all shape vitamin D needs and responsiveness. Vitamin D also relies on cofactors—such as magnesium and vitamin K_2_—to function optimally, yet most trials overlook this. Recognizing these interactions and individual differences is essential for sound public health guidance and for tailoring clinical care when appropriate.

## 5. Discussion

In the present work, evaluation of Hill’s criteria for vitamin D for nine diseases is reviewed: cancer, CVD, hypertension/BP, COVID-19, T1DM, T2DM, periodontal disease, multiple sclerosis, and dementia. While RCTs have had limited success in demonstrating that vitamin D supplementation reduces the risk of these and other diseases, Hill’s criteria analyses indicated that vitamin D reduced the risk of these diseases in a causal manner. The strongest evidence for vitamin D in Hill’s analyses is consistent findings in different populations, mechanisms, strength of association, and biological gradient. While confounding factors also affect risk of these diseases, they can generally be accounted for.

An important concern is whether serum 25(OH)D concentration or solar UVB dose indices are also indices for disease risk-modifying factors other than vitamin D. For example, it has been proposed that UV irradiance can increase serum NO concentrations, which can have anti-microbial and BP-reducing effects [204,205]. However, inspection of Table 1 in [204] finds that the dismissal of vitamin D effects for MS, hypertension, ischemic heart disease, T2DM, COVID-19, and all-cause mortality was based primarily on the failure of vitamin D RCTs or other vitamin D supplementation studies to result in reduced risk or clinical measures of disease. As already discussed in this review, most vitamin D RCTs have not been properly designed for vitamin D, which should be considered a nutrient, not a drug [40]. Thus, while NO may have an impact on the risk of some diseases, it cannot be considered an alternative hypothetical agent to vitamin D, especially for diseases such as cancer, which generally do not have seasonal variations in incidence [206].

Hill’s criteria analyses could also be conducted for other health outcomes, especially those with strong findings from PCS based on serum 25(OH)D concentration and/or vitamin D supplementation and known mechanisms. A good example is pregnancy and birth outcomes. A stratified randomized field trial of a prenatal vitamin D deficiency screening and treatment program was conducted in Iran [26]. Nine hundred pregnant women in two hospitals were included. The mean baseline 25(OH)D concentration in both centers was near 11 ng/mL. Those in one center were screened and supplemented to raise serum 25(OH)D concentrations above 20 ng/mL. Those in the other center were not treated. The incidence of gestational diabetes mellitus, pre-eclampsia, and preterm birth for those who achieved >20 ng/mL decreased by 50%, 60%, and 40%, respectively, in the screening site. The mechanisms of vitamin D during pregnancy are reasonably well known and include effects on placental function, neurodevelopment, and lung maturation and function [207].

### 5.1. Strengths and Limitations of Hill’s Criteria

The use of causal criteria in nutritional epidemiology was discussed in a 1999 review [109]. The five Hill’s criteria considered most important were consistency of association, strength of association, dose–response (biological gradient), biological plausibility, and temporality. No mention was made of experimental verification. Regarding consistency, there can be variations in how many and what fractions of studies have similar findings. Such information is often not stated clearly in the reviews. Strength of association can vary. The author suggested that 20% might be sufficient for public health guidance. Dose–response has several potential sources of error. For vitamin D, these include misclassification errors in which participants are categorized by serum 25(OH)D concentrations at a particular time. Misclassification could occur if participants only recently began supplementing with vitamin D since many chronic diseases take several years to develop. Also, unless the concentrations were seasonally adjusted, participants could be misclassified. Measurement error is another concern.

Assays for 25(OH)D concentrations have different responses [208]. As a result, the assays should be compared with liquid chromatography-tandem mass spectrometry (LC-MS/MS) methods through the Vitamin D External Quality Assessment Scheme (DEQAS) [209]. Another factor is genetic variation. Serum 25(OH)D is affected by genetic variations in the metabolic pathways from solar UVB dose and oral intake to 25(OH)D [17]. This effect is important when studies are based on solar UVB dose or oral intake but not if serum 25(OH)D is measured. Biological plausibility can be affected in vitamin D studies by other variables associated with oral vitamin D intake or serum 25(OH)D concentration. For example, fish and meat are important dietary sources of vitamin D [210], but they are also important variables for risk of diseases such as T2DM and CVD [211]. Satisfying temporality requires making sure that participants developed the disease after vitamin D status was determined. Thus, cross-sectional studies are generally not appropriate. In PCS, the first one or two years of data can be omitted to see if that changes the result, as done in the VITAL trial [60].

Weed also suggested that one of the lesser-used of Hill’s criteria, analogy, could play a more important role [212]. However, that requires having other vitamin D-health outcome relationships for which causality is established.

### 5.2. Evidence Gaps and Priorities for Future Research

Clinical researchers should follow the guidelines for nutrients as outlined by Heaney in 2014 [40]. The key steps include measuring serum 25(OH)D concentrations of prospective participants and enrolling mostly those with low concentrations, which would be those with VDD. The vitamin D_3_ doses should be high enough to increase concentrations to values which have been found through observational studies to be associated with significantly improved health outcomes. Serum 25(OH)D concentrations should be measured during the RCT both to check on compliance and to determine whether the vitamin D dose should be changed.

A good example of the importance of these two steps is found in an observational study of vitamin D supplementation of pregnant women in Iran [26]. There were 900 participants in each of the two hospitals. The mean 25(OH)D concentrations of the participants were near 11 ng/mL. Those in one hospital were monitored but not supplemented with vitamin D. Those in the other hospital were advised on how much vitamin D supplementation to take to achieve >20 ng/mL after each of several measurements of 25(OH)D. The result was that a significantly reduced risk of gestational diabetes, pre-eclampsia, and preterm delivery was found for those who achieved >20 ng/mL vs. those who did not.

The analysis of the data should be based on achieved 25(OH)D concentrations, not on intention to treat. A good example of why this approach is important comes from the Vitamin D and Type 2 Diabetes (D2d) study conducted at Tufts University. When the results were analyzed by intention to treat, the effect of vitamin D_3_ supplementation was not significant [213]. However, when analyzed in terms of achieved serum 25(OH)D concentration in the vitamin D treatment arm, it was found that increasing the concentration above 50 ng/mL vs. 20–30 ng/mL reduced the risk of conversion to diabetes mellitus, with a hazard ratio = 0.29 (95% CI, 0.17–0.50} [171].

A better understanding is required of how obesity affects the roles of vitamin D in reducing risk of disease. It is well-known that obesity is associated with lower 25(OH)D concentrations due to sequestering vitamin D in adipose tissue [214] as well as due to volumetric dilution [215]. Thus, it might be expected that obese individuals require higher vitamin D doses to achieve serum 25(OH)D concentrations similar to non-obese individuals. While that is correct, what is different is that doing so does not provide the same reduction in risk of disease as for the non-obese. For example, in the VITAL trial, taking 2000 IU/day vitamin D_3_ increased serum 25(OH)D from 33.3 to 45.9 ng/mL for those with BMI < 25 kg/m^2^, from 299.5 to 41.4 ng/mL for those with BMI from 25–30 kg/m^2^, and from 26.7 to 38.8 ng/mL for those with BMI > 30 kg/m^2^. Very little difference in risk of cancer would be expected for the three achieved 25(OH)D concentrations. However, while those with BMI < 25 kg/m^2^ had a 25% significantly reduced risk of cancer incidence, there was no significantly reduced risk for the higher BMIs.

An article in 2019 “critically summarize [d] emerging biological mechanisms linking obesity to cancer encompassing insulin resistance and abnormalities of the IGF-I system and signaling; sex hormones biosynthesis and pathway; subclinical chronic low-grade inflammation and oxidative stress; alterations in adipokine pathophysiology; factors deriving from ectopic fat deposition; microenvironment and cellular perturbations including vascular perturbations, epithelial–mesenchymal transition, endoplasmic reticulum stress and migrating adipose progenitor cells; disruption of circadian rhythms; dietary nutrients; factors with potential significance such as the altered intestinal microbiome; and mechanic factors in obesity and cancer [97]. A 2025 review stated “Obesity, characterized by a chronic inflammatory state, exacerbates this tumor-promoting environment through metabolic imbalances like insulin resistance, hyperglycemia, and dyslipidemia. These conditions stimulate oncogenic signaling pathways and reshape the tumor microenvironment. Obesity-associated cytokines, altered adipokines, and insulin-related growth signals synergistically enhance processes such as epithelial-to-mesenchymal transition (EMT) and matrix remodeling.” [216]. Another 2025 review noted that low vitamin D status in obesity may negatively impact adipose biology, leading to the question of whether raising serum 25(OH)D concentrations could lead to reductions in adiposity and reduced risk of cardiometabolic diseases [217].

## 6. Conclusions

This review demonstrates that, when evaluated through Bradford Hill’s criteria, accumulated evidence supports a causal role for vitamin D in reducing risk of multiple chronic diseases, including cancer, CVD, hypertension/BP, COVID-19, T1DM, T2DM, periodontal disease, multiple sclerosis, and dementia. The strongest support arises from consistent observational findings, biologically plausible mechanisms, dose–response relationships, and coherence with vitamin D’s nutrient-specific biology. Conventional randomized controlled trials have often failed to confirm these benefits due to methodological limitations, such as insufficient dosing, short durations, and reliance on assigned rather than achieved serum 25(OH)D concentrations. By contrast, well-designed ecological and prospective cohort studies, which reflect real-world variations in vitamin D status, provide more relevant and robust evidence. These findings underscore the need for nutrient-appropriate research frameworks, using observational studies instead of RCTs for evaluating nutrients, and cost-effective public health strategies that prioritize maintaining adequate serum 25(OH)D levels for disease prevention, as discussed in this review.

## Figures and Tables

**Table 1:** Representative examples of using Hill’s criteria to demonstrate causal dietary factor-disease relationships that are also in accordance with the present understanding. Thus, there is good precedent for applying Hill’s criteria for causality to vitamin D.

Outcome	Risk Factor	Hill’s Analysis	Present Understanding
CHD	Nuts	[47]	[48]
AD	Spirochetes	[49]	[50,51]
CKD	Dietary fiber	[52]	[53,54]
CVD	Anxiety	[55]	[56]
CVD	Red and processed meat	[57]	[58]
T2DM	Red and processed meat	[57]	[58,59]

Abbreviations: AD, Alzheimer’s disease; CHD, coronary heart disease; CKD, chronic kidney disease; CVD, cardiovascular disease; T2DM, type 2 diabetes mellitus.

**Table 2:** Vitamin D deficiency (VDD) rates in various countries.

Country	Age Range (yrs)	Population N	Years	Season	<VDD (ng/mL, %)	VDD (<20 ng/mL, %)	Reference
Africa	All	23 countries 21,676	1978–2019	A	<12, 17	34	[68]
Australia	>65	674 F 617 M	2011–2013	A	<12, F: 5; M: 3	F: 21; M: 8	[69]
Brazil	Elderly	20 studies	2000–2017	A		42	[70]
China	>65	2180	2000–2018	A	<12, 31	70	[71]
China	Adults	234,519	2000–2020	A	<12, 21	63	[72]
Czech Republic	51–90	General 57,984	2010–2020	A		34	[73]
Italy (south)	25–74	1200	2018–2019	A		57	[74]
Japan (Tokyo)		5518 Medical checkup	2019–2020	A		79	[75]
Saudi Arabia	>40	1767	2014–2017	A	<10, 10	45	[76]
UK	>18	Visiting PCP	2013–2015	W, S	<12, 38, 18		[77]
USA		General 3.44 million tests	2006–2011	W, S		<25 ng/mL, 38, 20	[78]
USA		General 3.8 million tests	2007–2009	W, S		32, 13 (39, 1) *	[79]

(*) vitamin D_3_ as opposed to vitamin D_2_ + vitamin D_3_; A, annual; PCP, primary care physician; S, summer; W, winter.

**Table 3:** Hill’s criteria for causality in a biological system used for vitamin D.

Criterion	
Strength of association	The greater the effect, the stronger the case.
Consistency	Similar findings made in different populations
Temporality	The exposure precedes the effect
Coherence with known science	There should be no conflicts with known science
Biological gradient	More pronounced effects as exposure to the agent increases
Plausibility	E.g., mechanisms to explain the effects
Experiment	Studies in which the agent is given and effects determined
Analogy	Are the relationships between agent and effect like what has been seen for other outcomes?
Accounting for confounding factors [109]	Using data for other risk-modifying factors in conjunction with the agent to determine if they also affect the observed outcome.

## Data Availability

No new data were created or analyzed in this study. Data sharing is not applicable to this article.
